# The Problem to a Solution: A Case of Local Anesthetic-Related Acute Pancreatitis

**DOI:** 10.7759/cureus.70681

**Published:** 2024-10-02

**Authors:** Jeffrey Robles, Dillon Rogando, Tara Ranjbar, Indraneil Mukherjee, Erika Clarke, Sourodip Mukharjee

**Affiliations:** 1 Surgery, Northwell Health Staten Island University Hospital/City University of New York School of Medicine, New York, USA; 2 General Surgery, Northwell Health Staten Island University Hospital/City University of New York School of Medicine, New York, USA; 3 General Surgery, Northwell Health Staten Island University Hospital, New York, USA

**Keywords:** acute pancreatitis, lidocaine reaction, lidocaine toxicity, local anesthetic systemic toxicity (last), necrotizing pancreatitis complication

## Abstract

Although rare, local anesthetic use has been associated with adverse central nervous system and cardiovascular adverse events. One complication is local anesthetic systemic toxicity (LAST), wherein the anesthetic agent inadvertently enters systemic circulation resulting in widespread inhibition of fast-gated sodium channels. Organs dependent on aerobic metabolism, such as the heart and brain, are especially susceptible to toxic injury resulting in cardiovascular collapse. Lipid emulsion therapy is a mainstay treatment of LAST; however, it may inadvertently cause lipid-induced necrotizing pancreatitis.

We present a 71-year-old female with a non-contributory past medical history who presented for elective open reduction and internal fixation (ORIF) of the right wrist after a fall onto the right hand one week prior. A supraclavicular brachial plexus block was planned to deliver regional analgesia for ORIF of the right wrist.

Shortly after the introduction of the local anesthetic agent, our patient became bradycardic and hypotensive. The patient's heart rate and systolic blood pressure remained in the low 45s and 50s, respectively, refractory to multiple doses of phenylephrine and ephedrine. Suspicion for LAST syndrome was high and lipid emulsion therapy was started. Once stabilized, the patient was transported to the ICU requiring manual ventilation, where she later reported flank pain. Labs drawn were notable for elevated triglyceride, lipase, and amylase levels of over 3000, 600, and 700, respectively. CT imaging confirmed acute necrotizing pancreatitis.

The patient's ICU stay was uncomplicated with stabilization of vitals and discharge 10 days later. A follow-up with gastroenterology (GI) was scheduled after two weeks. Since discharge, the patient noted intermittent abdominal aches. Magnetic resonance cholangiopancreatography (MRCP) and MRI were performed after liver function tests (LFTs) were found to be elevated during her follow-up appointment. MRI showed liquefaction necrosis of 80% of the pancreas, with a 14-centimeter fluid collection pushing on the distal common bile duct, causing extrinsic obstruction. MRCP revealed no stones. Advanced GI performed a transgastric cystogastrostomy with lumen apposing metal stent placement in the common bile duct, and drained the fluid from the obstructing cyst. Cytopathology came back as virtually acellular. Liver enzymes began to downtrend appropriately and the patient was discharged soon after. Follow-up at two and four days post-discharge confirmed resolution of symptoms.

## Introduction

Although rare, local anesthetic use has been associated with adverse central nervous system and cardiovascular adverse events. One complication of local anesthetic use is local anesthetic systemic toxicity (LAST), wherein the anesthetic agent inadvertently enters systemic circulation resulting in widespread inhibition of fast-gated sodium channels. Organs dependent on aerobic metabolism, such as the heart and brain, are especially susceptible to toxic injury resulting in cardiovascular collapse. Lipid emulsion therapy is the mainstay treatment of LAST [1]; however, it may inadvertently cause lipid-induced necrotizing pancreatitis due to high amounts of serum triglycerides. Management of lipid-induced pancreatitis is similar to the treatment of pancreatitis in general, requiring adequate intravenous hydration and pain control with analgesia. In the case of necrotizing pancreatitis, a course of broad-coverage antibiotics is also usually required to aid in wound healing and prevent disease progression. We present a case of LAST syndrome requiring lipid emulsion therapy, ultimately resulting in acute necrotizing pancreatitis.

## Case presentation

We present a case of a 71-year-old female with a non-contributory past medical history who presented for an elective open reduction and internal fixation (ORIF) of the right wrist after suffering a traumatic fall in her backyard one week prior. Plain radiographs of the right extremity, obtained on the day of the fall, demonstrated a comminuted fracture of the distal radius, which extended to the articular surface, and an ulnar styloid fracture. Preoperative evaluation of the patient was unremarkable and at the time of evaluation, the patient denied any symptoms of angina or dyspnea. Regional analgesia of the right upper extremity was planned using a supraclavicular brachial plexus block approach.

**Figure 1:**
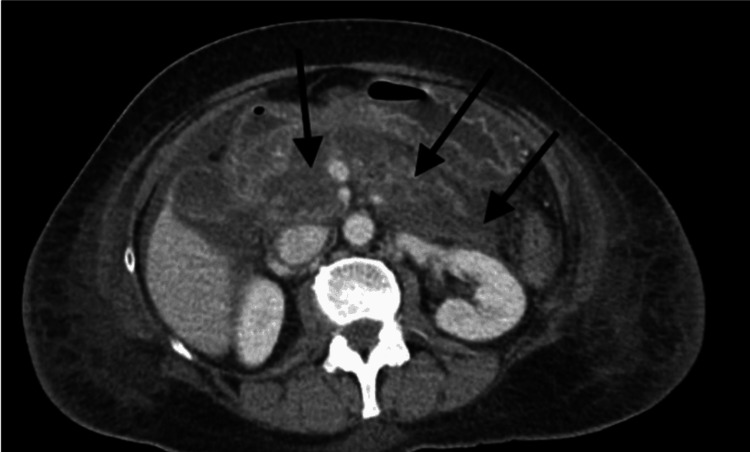
CT imaging confirming acute necrotizing pancreatitis. Arrows pointing to areas of acute edema and necrosis, confirming the presence of acute necrotizing pancreatitis hours after the use of intravenous lipid infusion.

The site was identified and sterilized, and 25 cc of 0.5% bupivacaine along with perioperative antibiotic was administered. Shortly after the introduction of the local anesthetic, the patient experienced bradycardia with a heart rate of 50 beats/minute. The patient was also found to be hypotensive with systolic blood pressure in the 70s. Despite the administration of a 10 microgram bolus dose of phenylephrine (Neo-synephrine), the patient remained hemodynamically unstable with no improvement in blood pressure. The patient’s heart rate dropped to 44 beats/minute after 10 minutes and diffuse ST elevations were noted. The patient’s blood pressure further dropped to 52/30 mm Hg, after which 10 milligrams of ephedrine was administered intravenously, and the propofol infusion was discontinued. Due to persistent and refractory hypotension, the patient received a second dose of 10 milligrams of ephedrine intravenously. There was high suspicion of LAST syndrome, and a decision was taken to begin lipid emulsion therapy. The patient received two boluses of 20% intralipids at a dose of 1.5 ml/kg, followed by a constant infusion of intralipids at a rate of 0.25 ml/kg/min. The patient was then intubated for airway protection and a right radial arterial line was placed for blood pressure monitoring. An arterial blood gas test was performed, and cardiac enzymes, a complete blood count, and a basic metabolic panel were ordered. Once stabilized, the patient was transported to the surgical intensive care (SICU) for ventilatory management and close monitoring. During transport to SICU, however, the patient required additional pushes of phenylephrine for hypotension with systolic blood pressure in the 60-70s. The patient's hemodynamic status improved upon arrival to the SICU. ECG was monitored throughout the transport. Due to agitated behavior and attempts to pull out her endotracheal tube and IV lines, a 2 mg IV push of midazolam was administered. A few hours later, the patient was still intubated but awake with complaints of epigastric pain. Labs revealed triglyceride levels over 3000, lipase over 600, and amylase over 700. Follow-up CT confirmed the presence of acute pancreatitis with areas of edema and necrosis, consistent with the diagnosis of acute necrotizing pancreatitis (Figure 1).

**Figure 2:**
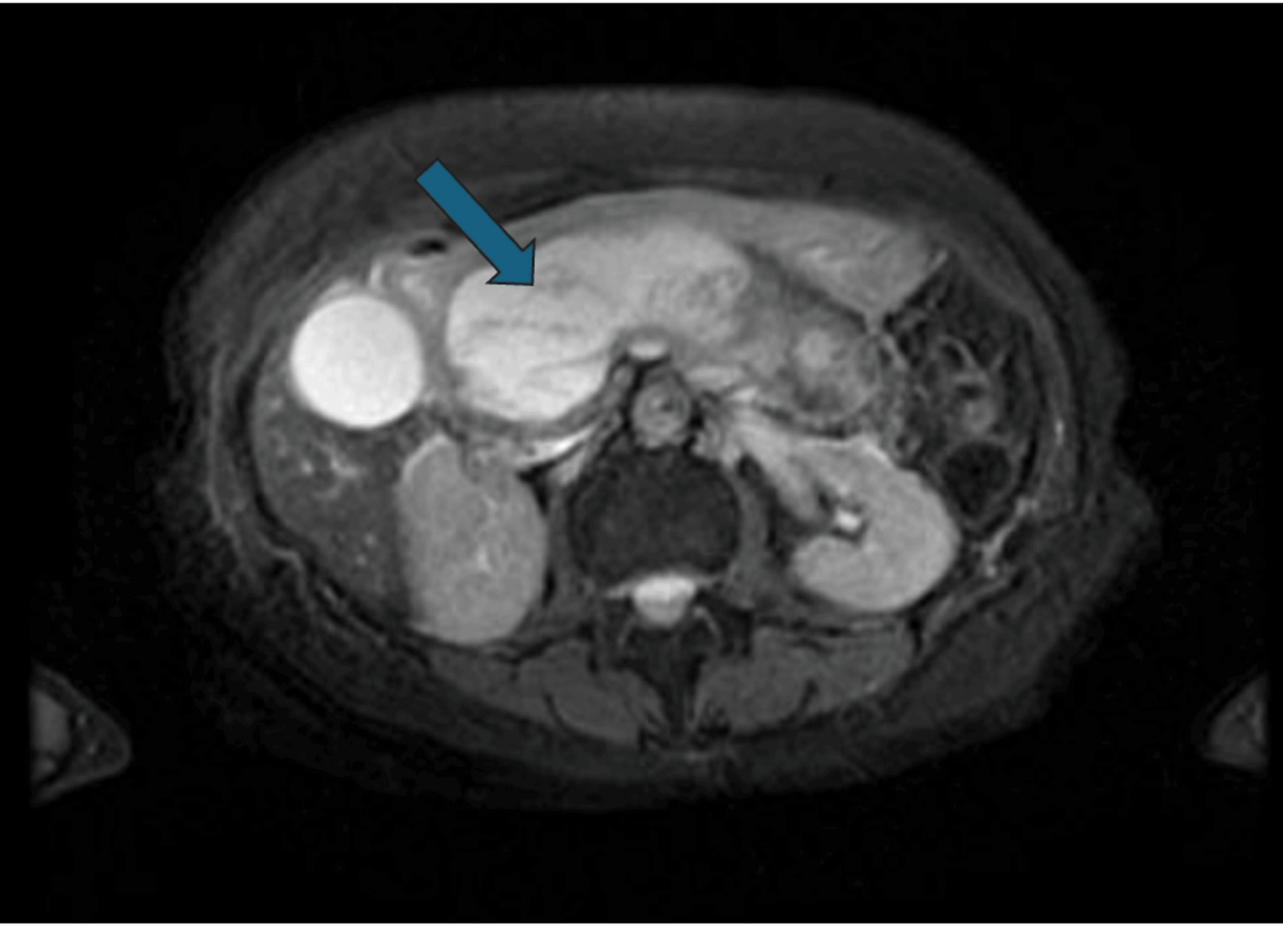
Walled-off fluid collection in the pancreas. Abdominal MRI showing a walled-off 14 cm pancreatic fluid collection, obstructing the common bile duct.

The patient's acute necrotizing pancreatitis was managed with intravenous hydration and pain control. Her serum triglyceride, lipase, and amylase levels normalized back to baseline the week following her brachial plexus block and was appropriately discharged. At her follow-up visit with GI two weeks later, magnetic resonance cholangiopancreatography (MRCP) was performed for elevated liver enzymes accompanied by complaints of abdominal pain. MRCP revealed liquefaction necrosis of 80% of the pancreas, with a 14-centimeter fluid collection pushing on the distal common bile duct (Figure 2). Transgastric cystogastrostomy with lumen-apposing metal stent placement in the common bile duct was performed. The patient was discharged that evening, reporting resolution of her abdominal symptoms at two and four days post discharge.

## Discussion

Local and regional anesthesia are highly effective interventions to provide analgesia in the setting of minor surgical procedures while avoiding the potential complications of general anesthesia and consequent intubation and mechanical ventilation. Several regional anesthetic techniques have been developed, including neuraxial anesthesia and peripheral nerve blocks, which have been shown to provide superior pain control as compared to general anesthesia in a variety of settings. In the case of our patient, a supraclavicular brachial plexus block was used to deliver local anesthetic in the right upper extremity for an ORIF of the wrist fracture.

The use of local anesthesia for pain control has come a long way since 1884 when the first clinical operation under local anesthesia was performed using cocaine administration [1]. Although effective as an anesthetic, cocaine abuse and misuse ran rampant among patients and healthcare workers [1]. Soon the dangerous effects of cocaine toxicity were noticed, thus necessitating the need for alternative local anesthetic medications. Between the years of 1891 and 1930, newer amino ester local anesthetics such as tetracaine, benzocaine, holocaine, orthoform, eucaine, and tropocaine were discovered. The amino amide local anesthetics such as nirvaquine, procaine, lidocaine, mepivacaine, efocaine, prilocaine, etidocaine, chloroprocaine, cinchocaine, articaine, and bupivacaine were also discovered around this time, most notably between the years of 1898 and 1972 [1]. Although substantially less toxic than cocaine, the use of these newer local anesthetics was associated with varying central nervous system and cardiovascular depression and/or excitation.

Amide and ester anesthetic agents inhibit voltage-gated sodium channels, which prevents the influx of sodium across neuronal cell membranes resulting in its analgesic effect. In the absence of sodium ion influx, action potentials across sensory neurons are unable to be conducted [2]. Local anesthetics have the greatest affinity for receptors within sodium channels during their active or inactivated phase (depolarized or hyperpolarized phase, respectively) than when in their resting phase, thus having a greater effect on those neuronal fibers that have higher rapid firing rates. Additionally, smaller neuronal fibers are more susceptible than larger fibers to local anesthesia due to their smaller number of sodium channels. For these reasons, smaller and rapid-firing autonomic fibers, followed by sensory and somatic fibers, are most susceptible to the analgesic effects of local anesthesia [2]. This is supported by the order in which neuronal function returns to patients after local anesthesia administration. As patients recover from spinal anesthesia, for example, first their voluntary motor function returns, followed by sensation, and lastly autonomic control of their bladder [2].

In general, amide anesthetic use is preferred over esters because of their decreased likelihood of causing hypersensitivity reactions and lower incidence of toxicity [3]. Unlike amide anesthetics, ester anesthetics contain metabolite p-aminobenzoic acid (PABA), which is a known allergen in susceptible individuals. Although amide anesthetics do not contain this metabolite, the preservatives used to prepare amide-type anesthetics can also be metabolized into PABA or PABA-like compounds and induce a similar allergic response [4].

The adverse effects of local anesthetic use are rare and are generally self-limiting. Some common reactions include headache, dizziness, pain around the insertion point, tingling sensation around the insertion point, and swelling of the insertion point. Effects of more complicated procedures requiring local anesthesia, such as epidural analgesia, may include loss of bladder control and temporary numbness. A potentially life-threatening adverse effect of local anesthetic use, however, is LAST [5], with an incidence rate of approximately 25 per 10,000 nerve blockades [5]. Patient populations with an increased risk for LAST include geriatric populations, neonates and infants under four months, Crohn's disease patients, and patients with pre-existing heart arrhythmias [5]. Additionally, the use of intercostal and intravenous regional techniques in these populations carries the highest risk for LAST. Most cases of LAST occur due to inappropriate intravascular administration of local anesthesia or improper dosing; however, this was not the case with our patient. Although the exact mechanism behind local anesthetic-induced systemic toxicity has not been fully elucidated, it is believed that local anesthetics inhibit components of the oxidative phosphorylation pathway. By doing so, organs dependent on aerobic metabolism such as the heart and brain, are especially susceptible to local anesthetic toxicity [5].

Central nervous system (CNS) findings are usually the only findings present in LAST, such as confusion, dizziness, agitation, drowsiness, auditory changes, tinnitus, perioral numbness, and dysarthria. Severe complications of CNS toxicity include seizure, coma, and respiratory arrest. Cardiac complications associated with LAST include dyspnea, hypotension, and bradycardia. More severe complications such as bradyarrhythmia, asystole, and ventricular fibrillation may occur [5]. Although all anesthetics carry the potential to cause CNS depression, bupivacaine is unique in that it exhibits a greater potential for causing direct cardiac effects than other local anesthetics [2]. In consideration of our patient’s rapid onset bradycardia and hypotension after bupivacaine administration, clinical suspicion for LAST was high.

When LAST is suspected, initial management should focus on airway management and circulatory support. Immediate ventilation and oxygenation should be performed to prevent hypoxia and acidosis, reducing the likelihood of progression to seizure. In the event of seizures, immediate administration of a benzodiazepine is warranted [5]. Further hemodynamic management focuses on restoring cardiac output and preventing cardiovascular collapse. Small doses of epinephrine (less than 1 microgram/kg) are preferred in the setting of local anesthetic-induced cardiac arrest. Vasopressin, calcium channel blockers, and b-adrenergic receptor blockers are contraindicated. If ventricular arrhythmias were to occur, amiodarone would be the first line of management as procainamide may worsen the existing toxicity [5].

Recent case studies have recommended lipid emulsion therapy as soon as prolonged seizure activity or local anesthetic-induced arrhythmias are suspected. By infusing the patients with lipids, the lipid-soluble local anesthetic may be drawn out of the cardiac tissue, improving cardiac conduction, contractility, and coronary perfusion [5]. If cardiac stability has not been achieved after epinephrine and lipid emulsion therapy, cardiopulmonary bypass is recommended until the local anesthetic has been cleared [5]. Following the recommended bupivacaine toxicity protocol, our patient was intubated, and epinephrine was administered followed by lipid emulsion therapy. Soon her cardiovascular status improved, and the patient was transferred to the SICU, where she had complaints of flank pain. At that time, labs were notable for elevated triglyceride, lipase, and amylase levels of over 3000, 600, and 700 respectively. CT imaging revealed acute pancreatitis with regions of edema and necrosis, leading our team to make a diagnosis of lipid-induced acute necrotizing pancreatitis.

The pathophysiology of triglyceridemia-induced acute pancreatitis is not exactly known, but it is believed that pancreatic lipase is released in response to triglyceridemia, which produces high levels of free fatty acids in the pancreas. These free fatty acids induce an inflammatory response resulting in premature pancreatic enzyme activation, free radical formation, edema, and necrosis [6]. Other potential causes of pancreatic inflammation and premature activation of pancreatic enzymes include gallstone disease, alcoholism, trauma, sepsis/infection, certain medications, certain cancers, and chronic cigarette smoking [7]. Severe consequences of acute pancreatitis include systemic inflammation, which may result in multi-organ dysfunction such as acute respiratory distress syndrome, acute renal failure, and myocardial ischemia.

The management of hypertriglyceridemia-induced acute necrotizing pancreatitis is like the management of acute pancreatitis in general. Aggressive hydration with IV fluids, nothing per mouth (NPO) until the abdominal pain and nausea resolve, a small low-fat diet, and pain control with nonsteroidal anti-inflammatory drugs and opioid analgesic use are routine [8]. In addition to this, serum triglyceride levels should be lowered, with our patient's serum triglyceride levels lowering from 3180 to 118 within the first 48 hours, and an outpatient serum triglyceride level of 89 during her follow-up visit two weeks after discharge. Although insulin therapy and plasmapheresis can be used to rapidly lower serum triglyceride levels in the inpatient setting, neither intervention was performed in our patient's case. Antibiotic use is not routinely required, but in patients such as ours with risk factors like older age and extensive involvement of disease, coverage with a carbapenem is indicated. After appropriate fluid hydration, pain management, and resumption of oral feeding status, the patient was discharged on a two-week course of meropenem. The patient did not follow up with the surgery team post-discharge but was admitted for an uncomplicated elective cholecystectomy a year later due to gallstone disease.

## Conclusions

This case presentation is to review a rare presentation of LAST syndrome, complicated by acute necrotizing pancreatitis. Consent was attained from the patient to use her hospital stay for the purpose of educational literature. It is important for physicians to understand the adverse events associated with both local anesthetic use and lipid emulsion therapy. Although rare, both LAST and acute necrotizing pancreatitis are potentially lethal complications of surgical procedures. Although the exact etiology of LAST is still being explored, hypertriglyceridemia is a well-known cause of pancreatitis and should be worked up in patients who receive lipid emulsion therapy. Intercostal and intravenous regional techniques carry the highest risk of LAST, with certain populations being more susceptible to LAST such as neonates and infants under four months, geriatric patients, and Crohn's disease patients. As such, performing close monitoring after anesthesia injection and use of non-intravenous peripheral nerve block techniques in high-risk populations should be considered when administering local anesthesia. The mainstay treatment of acute necrotizing pancreatitis secondary to hypertriglyceridemia is with serum triglyceride level control, pain management, fluid resuscitation, and use of antibiotic therapy if indicated.
